# Best practice approaches to outpatient management of people living with Parkinson’s disease during the COVID-19 pandemic

**DOI:** 10.1007/s00702-022-02484-7

**Published:** 2022-03-04

**Authors:** Antonia F. Demleitner, Andreas W. Wolff, Johanna Erber, Friedemann Gebhardt, Erica Westenberg, Andrea S. Winkler, Susanne Kolbe-Busch, Iris F. Chaberny, Paul Lingor

**Affiliations:** 1 Department of Neurology, School of Medicine, University Hospital München rechts der Isar, Technical University of Munich, Munich, Germany; 2 Department of Internal Medicine II, School of Medicine, University Hospital München rechts der Isar, Technical University of Munich, Munich, Germany; 3 Department of Hospital Hygiene, School of Medicine, University Hospital München rechts der Isar, Technical University of Munich, Munich, Germany; 4 Center for Global Health, School of Medicine, University Hospital München rechts der Isar, Technical University of Munich, Munich, Germany; 5 Centre for Global Health, Institute of Health and Society, School of Medicine, University of Oslo, Oslo, Norway; 6 Institute of Hygiene, Hospital Epidemiology and Environmental Medicine, Leipzig University Hospital, Leipzig, Germany; 7 DZNE, German Center for Neurodegenerative Diseases, Munich, Germany; 8 Munich Cluster for Systems Neurology (SyNergy), Munich, Germany

**Keywords:** COVID-19, Parkinson’s disease, Outpatients, Management, Best practice

## Abstract

The prevalence of Parkinson’s disease (PD) is rising, rendering it one of the most common neurodegenerative diseases. Treatment and monitoring of patients require regular specialized in- and outpatient care. Patients with PD are more likely to have a complicated disease course if they become infected with severe acute respiratory syndrome coronavirus type 2 (SARS-CoV-2). Regular in-hospital appointments place these patients at risk of exposure to SARS-CoV-2 due to travel and contact with other patients and staff. However, guidelines for the management of outpatients with PD during times of increased risk of infection are currently lacking. These are urgently needed to conduct risk–benefit evaluations to recommend the best medical treatment. This article discusses best practice approaches based on the current literature, as suggested by the multidisciplinary Network of University Medicine (NUM) in Germany. These include measures such as mask-wearing, hand hygiene, social distancing measures, and appropriate testing strategies in outpatient settings, which can minimize the risk of exposure. Furthermore, the urgency of appointments should be considered. Visits of low urgency may be conducted by general practitioners or via telemedicine consultations, whereas in-person presentation is required in case of moderate and high urgency visits. Classification of urgency should be carried out by skilled medical staff, and telemedicine (telephone or video consultations) may be a useful tool in this situation. The currently approved vaccines against SARS-CoV-2 are safe and effective for patients with PD and play a key role in minimizing infection risk for patients with PD.

## Introduction

Due to an aging population and ever-improving treatment options, the number of patients with Parkinson’s disease (PD) is steadily increasing (Dorsey et al. 2018). This makes PD one of the fastest growing neurodegenerative diseases worldwide (Deuschl et al. 2020) which also requires substantial resources for specialized in- and outpatient care. Due to the chronic nature of the disease, PD patients require regular outpatient consultations to monitor disease progression, to assess social indicators of coping, and to adjust therapy (Richter et al. 2019). PD patients are also more likely to be hospitalized than peers of their age, e.g., due to a sudden worsening of symptoms as a result of an infection (Huse et al. 2005). The coronavirus disease 2019 (COVID-19) pandemic caused by the severe acute respiratory syndrome coronavirus type 2 (SARS-CoV-2) continues to challenge healthcare systems worldwide. In many ways, it presents a more significant threat to patients with PD, especially as emerging variants of concern (VOC) further increase the contagiousness of the virus (Davies et al. 2021). If these patients are infected, the current treatment options for COVID-19, including IL6-blockers, neutralizing antibodies, and systemic corticosteroids, are still insufficient for preventing a severe course of disease (Rodriguez-Guerra et al. 2021), even if recent data on the ribonucleoside analog molnupiravir are promising (Mahase 2021). Therefore, best practice approaches for the management of outpatients with PD are urgently needed to control the infection risk for this vulnerable patient group. As part of Germany’s national pandemic response, the Network of University Medicine (NUM) has developed best practice approaches for the management of outpatients in a pandemic situation that take into consideration the vulnerabilities and special medical needs of this patient population. This article will present a summary of the key issues that should be considered in the management of PD patients in the context of the current and potential future pandemics.

## Patients with PD are at an increased risk for a complicated COVID-19 disease course

Older age and male sex are important risk factors for a severe course of and/or fatality after COVID-19 infection, as are diabetes, obesity, chronic obstructive pulmonary disease, dementia, or previous neurological disease (Williamson et al. 2020; Covino et al. 2020; Grasselli et al. 2020; Zhou et al. 2020; Bonanad et al. 2020; Ko et al. 2021). Pulmonary disease, obesity, and hypertension have also been specifically identified as risk factors for a severe COVID-19 disease course in PD patients (Chambergo‐Michilot et al. 2021; Fasano et al. 2020c; Huber et al. 2021). Although the risk of a complicated COVID-19 disease course is increased for people with older age and associated arterial hypertension, this risk is even higher in PD patients, especially with longer disease duration, than in non-PD patients (Fasano et al. 2020b, c; Orlando et al. 2021; Chambergo‐Michilot et al. 2021). PD patients may also have a higher mortality risk from COVID-19 infection per se (Zhang et al. 2020b; Rutten et al. 2020; Grasselli et al. 2020; Parihar et al. 2021). This calls for critical evaluation and planning of outpatient visits to provide sufficient care for these patients during the pandemic without placing them at an unnecessary risk of infection.

## Risk of exposure is increased by in-person appointments

Healthcare delivery, regardless of the setting, carries a risk of exposure to individuals who have an acute SARS-CoV-2 infection; the magnitude of the risk also depends on the true local incidence for SARS-CoV-2. The potential risks for exposure can be found not only in the healthcare setting itself, but also along the patient’s journey to an in-person appointment. For example, when taking public transportation, there is prolonged contact with potentially infectious people in what are usually poorly ventilated areas, so the risk of exposure to SARS-CoV-2 would increase accordingly. Once a patient has arrived at the treatment facility, vaccinated personnel can still potentially transmit the virus even if all recommended preventative measures have been followed, due to the emergence of highly transmissible or immune escape variants (Harris et al. 2021; Bailly et al. 2021; Kuzmina et al. 2021). Additionally, the facilities themselves, namely hospitals and outpatient clinics, have a concentration of potentially infectious patients and caregivers, which is why the implementation of entry-screening guidelines is vitally important. By assessing incoming patients and caregivers for typical COVID-19 symptoms and recent high-risk contacts, the risk of unscreened infectious individuals entering vulnerable areas can be reduced, but never completely abolished (Wang et al. 2020). Implementation of rapid testing strategies that include outpatients should therefore also be considered.

Due to the risks of infection in medical facilities (Richterman et al. 2020; Arons et al. 2020; Rickman et al. 2021), solutions must be implemented to mitigate the risk of in-person appointments. These include, but are not limited to, reducing exposure times in both waiting areas and examination/treatment rooms, as well as strictly adhering to standard practices for infection control, which encompass hand hygiene, respiratory hygiene (cough and sneeze etiquette), the use of medical face masks, and a no-handshake policy. In addition, the frequency of appointments should be adjusted to avoid prolonged gatherings in waiting areas. In model calculations for a room with four patients, of whom one is infectious, regular active ventilation reduces the individual risk of an infection from 36 to 15%. Additional measures, such as wearing a face mask, reduce the individual risk to as low as 0.8%, depending on the quality of the mask. A meta-analysis, which included studies from previous coronavirus outbreaks as well as the current pandemic, suggested that FFP2 (Filtering Face Piece Class 2)/KN95 respirator masks might provide better protection compared to regular face masks (Chu et al. 2020). In rooms where active ventilation is not possible, facilities should consider the usage of high-volume high-efficiency particulate air (HEPA) filtering devices (Lelieveld et al. 2020).

Special attention should be paid to patients returning from high-incidence regions, those who have been in contact with infected people and those recovering from a COVID-19 infection, in case of prolonged viral shedding and asymptomatic infections. Rigorous screening and testing concepts should also be implemented for patients coming from nursing homes, as virus transmission from asymptomatic patients has previously been linked to outbreaks (Byambasuren et al. 2020; Borras-Bermejo et al. 2020; Giri et al. 2021). Whenever possible, the number of people present at in-person appointments should be kept to a minimum and additional nursing personnel should only accompany patients if absolutely necessary. To further mitigate exposure risk, the duration of the visit should also be kept as short as possible.

## Assessment of urgency of in-person appointments

Particular attention must be given to the urgency of a visit when scheduling outpatient visits for PD patients during a pandemic situation (Table 1) (Simonet et al. 2019). For example, routine visits can be considered low urgency visits if the patient is clinically stable. Here, it might be appropriate to refer the patients to the primary care physician/general practitioner (GP), whom the patient will also likely consult for other reasons, for performing laboratory tests or issuing follow-up prescriptions (Kearon and Risdon 2020). A visit may be classified as moderately urgent if a patient is experiencing a worsening of symptoms that limit activities of daily life and therefore require therapeutic intervention, but do not require immediate action, such as worsening of fine motor skills, continued reduction in walking distance, dysphonia, increasing cognitive impairment, mild depressive symptoms, or sleep disturbances. Lastly, severe worsening of symptoms, e.g., akinetic crisis, acute delirium, or acute deterioration due to other medical conditions such as infection or exsiccosis, is considered highly urgent and requires immediate attention and, in many cases, hospitalization (Gerlach et al. 2011; Woodford and Walker 2005). Patients with advanced PD may also need urgent attention due to the malfunction of device-aided therapies such as deep brain stimulation (DBS) or pump therapies (Miocinovic et al. 2020; Fasano et al. 2020a). Here, a specialized team experienced in the treatment of advanced PD stages must be involved.

**Table 1:** Exemplary reasons for an outpatient clinic visit in patients with PD, stratified by urgency level

Low urgency	Moderate urgency	High urgency
Routine visits without clinical worsening	Worsening of fine motor skills	Akinetic crisis
Renewal of prescriptions	Reduction in walking distance	Acute delirium
Routine control of DBS or pump therapy	Progressive cognitive impairment	Exsiccosis, infection
Routine laboratory controls	Adaptation of DBS or pump therapy parameters	Device dysfunction of DBS aggregate or pump therapy
	Mild depressive symptoms	Acute depression
	Dysphonia	Falls, trauma

Frequently, an assessment of urgency can be performed prior to in-person visits by means of video or telephone consultations between the patient, caregiver and/or family member and the treating neurologist or trained medical personnel from the outpatient clinics, such as a trained Parkinson nurse, nurse practitioner, or physician assistant (Fasano et al. 2020a). In this way, the risk of infection can be reduced for this vulnerable patient group. Another desirable option, which is not yet widely established, would be to remotely adjust DBS electrode settings with simultaneous video monitoring of therapeutic effects (Cerroni et al. 2020). Initial studies show a high level of satisfaction with the settings adjusted in a video consultation among patients in a Chinese cohort (Zhang et al. 2020a). However, if there is any doubt about the urgency of a visit, an immediate in-person appointment should be planned.

In a pandemic situation that is not completely controlled, such as in the case of exponential growth or the appearance of novel virus variants with yet unknown properties, the frequency of outpatient appointments may need to be rapidly adjusted. It is therefore recommended that clinics set up an appointment system that can inform patients quickly and easily about the date and modality of their next outpatient visit. Digital scheduling solutions can save time for outpatient clinic staff and give patients more flexibility in choosing and rebooking visits.

## Telemedicine approaches should be implemented whenever possible

However, not all PD patients are comfortable with digital solutions for scheduling and may prefer the conventional methods for making appointments and contacting their clinicians. Virtual appointments have been shown to have no negative impact on patients’ quality of life (Beck et al. 2017; Hatcher-Martin et al. 2020), and there are clear advantages in efficiency, feasibility, and convenience, with a lower risk of exposure for both patients and practitioners. Unfortunately, telemedicine appointments are frequently financially less attractive to hospitals and are not always covered by health insurance in many countries (Dorsey et al. 2020; Patterson 2020). However, PD patients are generally willing to test telemedicine as an alternative to the traditional visits, and the usage of telemedicine approaches increased during the pandemic (Del Prete et al. 2020; Hassan et al. 2020). Currently, one of the most widely practiced approaches for remote physician–patient interaction is a telephone consultation, which has minimal technical requirements on both sides and can be implemented quickly. Several groups have described the success of using video rounds to provide a focused neurological examination, with some assistance from caregivers (Grossman et al. 2020; Fasano et al. 2020a). Smartphone applications (apps) increasingly enable patients to record symptoms themselves, although this greatly depends on the patient’s capabilities and individual access to technical solutions (Miele et al. 2020). Particularly in the older patient population, clear limitations remain due to digital illiteracy, which may lead to difficulties in setting up video appointments or recording data in an app. Caregivers will need to assist in enabling access to these opportunities.

## Vaccinations against COVID-19 are safe and should be considered for all PD patients

Apart from the aforementioned measures of infection control, vaccinations represent one of the most important pillars of the prevention strategy. Several mRNA-, vector-, and inactivated-virus-based vaccines against SARS-CoV-2 have been approved by the WHO and have proven safe and efficacious for reducing the risk of infection and complicated course of COVID-19 disease (Polack et al. 2020; Baden et al. 2021; Voysey et al. 2021; Sadoff et al. 2021; Tanriover et al. 2021; Al Kaabi et al. 2021). Empirical evidence has also shown reduced transmission from breakthrough infections after vaccination (Shah et al. 2021). So far, there have been no data published that found any negative long-term consequences after administration to the at-risk population of PD patients (Bloem et al. 2021). Although the incidence is very rare, female patients and those under the age of 60 have a higher risk of cerebral venous thrombosis when vaccinated with vector-based vaccines, and therefore, mRNA-vaccines may be more appropriate in these groups (Greinacher et al. 2021). Nevertheless, there is currently no evidence that PD patients suffer more frequent or more dangerous side effects from currently approved vaccines. The Scientific Issue Committee of the International Parkinson and Movement Disorder Society therefore recommends that all patients with PD be vaccinated, especially those who are at increased risk for a lethal course of infection due to their advanced age or comorbidities (Bloem et al. 2021).

## Conclusion

**Fig. 1:**
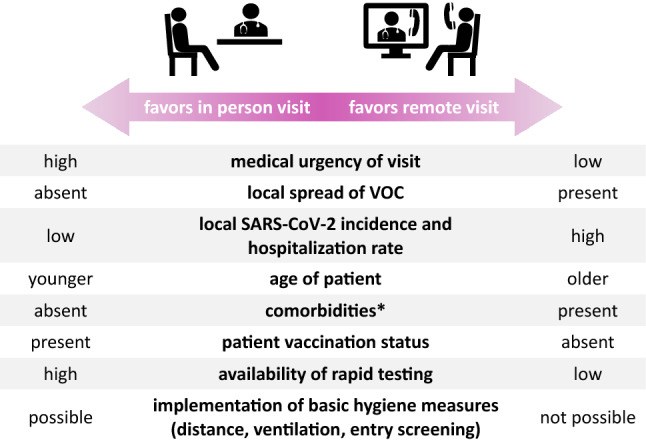
Factors to consider when deciding whether an in-person visit or a telemedicine visit is preferred. *VOC* variant of concern. *Comorbidities predisposing to worse outcome in COVID-19, such as obesity, diabetes mellitus, chronic lung or kidney disease, cardiovascular disease, immune deficiency, or cancer

So far, best practice approaches on the management of outpatients with PD have been lacking. Therefore, an interdisciplinary team of the Netzwerk Universitätsmedizin (NUM) has developed recommendations based on the current literature as part of Germany’s pandemic response. In summary, multiple factors should be considered when planning outpatient visits for patients with PD in the current pandemic situation. PD patients are at increased risk of complicated and fatal courses in the event of a SARS-CoV-2 infection. As outpatient clinic visits pose an increased risk of infection, the urgency of an in-person appointment for these patients should be carefully weighed against infection prevention. Other factors, such as the severity of the current local pandemic situation and the patient’s individual risk profile, as well as their digital literacy, need to be considered as well (Fig. 1). A decision about whether a visit should be conducted in person or can be performed via telemedicine should only be made upon careful consideration of these factors. The implementation of such best practice approaches will facilitate the management of PD outpatients during the current COVID-19 pandemic and contribute to better preparedness for future pandemic situations.
